# Supporting the investigation of health outcomes due to airborne emission by different approaches: current evidence for the waste incineration sector

**DOI:** 10.1007/s11356-024-34989-x

**Published:** 2024-09-24

**Authors:** Francesco Di Maria, Federico Sisani, Daniela Cesari, Elza Bontempi

**Affiliations:** 1 Department of Engineering, University of Perugia, Via G. Duranti 93, 06125 Perugia, Italy; 2 National Research Council, Institute of Atmospheric and Climatic Sciences Institute, S.P Lecce-Monteroni Km 1.2, Lecce, Italy; 3 INSTM and Chemistry for Technologies Laboratory, University of Brescia, Via Branze 38, 25123 Brescia, Italy

**Keywords:** Environmental impact, Epidemiologic survey, Life cycle assessment (LCA), Human health, Municipal solid waste incineration (MSWI), Oxidative potential

## Abstract

Life cycle assessment (LCA) along with a survey on epidemiologic and oxidative potential studies was used for analysing the current evidence of the impact of airborne emissions from municipal solid waste incineration (MSWI) on human health. The correspondence among investigated health outcomes and pollutants was discussed based on the Chemical Abstract Service (CAS) and the International Agency for Research on Cancer (IARC). LCA indicated the ability of MSWI in avoiding human health impact, about − 2 × 10^−4^ DALY/tonne together with avoided emissions of particulate matter (PM) and resource depletion, about − 2.5 × 10^−3^ kg Sb_eq_/tonne and about − 0.11 kg PM_2.5_ eq/tonne, respectively. Positive emissions were detected for greenhouses (about 900 kg CO_2eq_/tonne) and ecotoxicity (about 15,000 CTUe/tonne). Epidemiologic studies performed on population exposed to MSWI reported quite contrasting results. In some of these, hazard ratio (HR) ranging from about 0.7 to 2.2 was reported concerning the incidence of stomach, liver, breast and bladder cancer. Larger agreement was detected concerning the incidence of larynx and lung cancer with HR ranging from about 1 to about 2.6. Direct causal nexuses were not definitively identified. Oxidative potential of PM was characterized by a high Pearson correlation > 0.8 to the presence of CrVI, Cu and Zn. These heavy metals were also identified by both CAS and IARC as toxic (i.e. Cu and Zn) and cancerous (i.e. CrVI) substances affecting the organs of both respiratory and digestive apparatus. In general, even if more research is necessary, LCA, oxidative potential and the epidemiologic survey results showed a high level of accordance. This suggests their integrated exploitation for supporting the investigation of both direct and indirect consequences on environment and health related to waste incineration for both retrospective and predictive studies.

**Supplementary Information:**

The online version contains supplementary material available at 10.1007/s11356-024-34989-x.

## Introduction

The European Union (EU) legislation of waste management (WFD, 2008) imposes that recovery is the priority operation to be performed on those not reusable and not recyclable waste. Energy recovery by municipal solid waste incineration (MSWI) facilities, operated at high energy efficiency, resulted in the most adopted solution for this aim with about 500 facilities processing about 30% of the whole municipal waste generated in the EU area (ISPRA 2018). The main benefits from MSWI are represented by the replacement of fossil fuels and by the reduction of landfill needs and related emissions (Di Maria and Sisani 2018; Di Maria et al. 2018a; Khan et al. 2022; Roy et al. 2022).

The ability of MSWI in recovering energy was largely documented in literature (Di Maria et al. 2018b; ISWA 2016) as its ability in reducing the mass of the landfilled waste up to > 80% w/w (Czop and Ła´zniewska-Piekarczyk 2020; Di Maria and Sisani 2018).

One of the main environmental concerns for MSWI was represented by airborne emissions (Domingo et al. 2020; Tait et al. 2020; Anand et al. 2021; Gómez-Sanabria et al. 2022) leading the EU to the adoption of specific and stringent legislation for this sector (EC 1996, 2010). The effect of the legislation coupled with the technological evolution led, in the last 30 years, to a significant drop of the whole emission from MSWI up to > 90% (ISPRA 2019, 2021a).

**Fig. 1:**
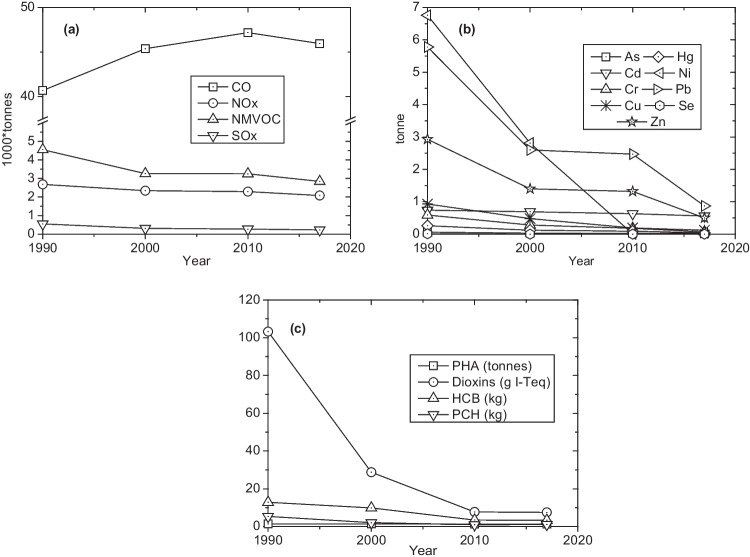
Trend of the whole macropollutant (**a**), micropollutant (**b**) and organic pollutants (**c**) emitted from waste incinerated in Italy from 1990 to 2017 (ISPRA 2019)

From 1990 to 2017, for the Italian context, generalized and significant reduction of the whole amount of the monitored emissions from MSWI was detected (ISRPA, 2019) (Fig. 1). All this occurred even if the amount of waste incinerated increased from about 1,800,00 tonnes/year (1990) to about 5,300,000 tonnes/year (2017).

**Fig. 2:**
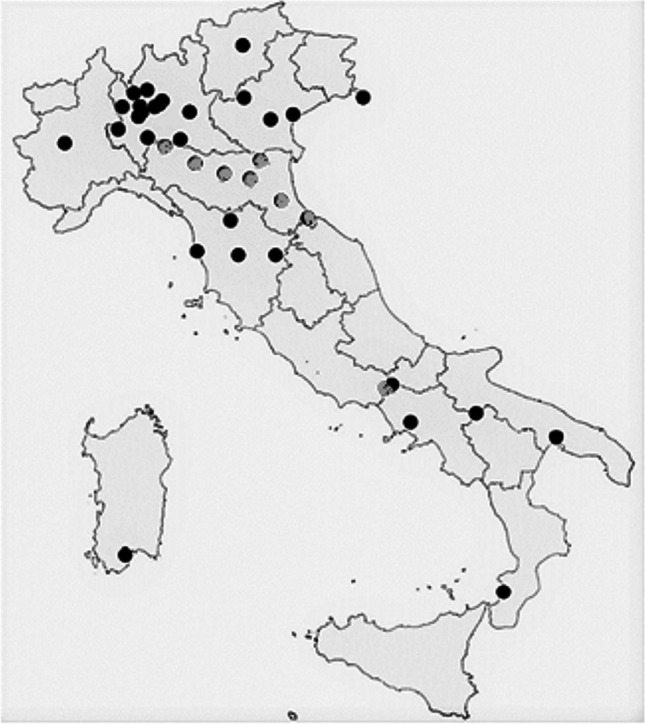
Italian municipal solid waste incinerators facilities (grey = involved in epidemiologic studies)

Figure 2 reports the geographical distribution of the current MSWI facilities operating in Italy with 26 plants located in Northern Italy, 5 in Central Italy, and 6 in Southern Italy (ISPRA, 2021b).

Concerning the consequences on human health, the cohort retrospective epidemiologic studies performed for MSWI in the EU area were mainly focused on the investigation of congenital malformations, specific tumours and birth defect health outcomes also based on hospital admissions and mortality (Ancona et al. 2013; Elliott et al. 1996; Goria et al. 2009; Ranzi et al. 2011). Despite a quite diffused detection of correlations among health outcomes and MSWI, definitive casual nexus was not demonstrated with risk ratios (RR) ranging from 0.15 to 2.3 depending on the specific disease investigated. This was mainly a consequence of some methodological flaws disregarding, e.g. confounding factors (lifestyle, social conditions, technology description, other sources of emissions) and other relevant aspects (Gullis and Fujino 2015). In another cohort study retrospective, Ghosh et al. (2019) investigated the effects of UK MSWI exposure on reproductive outcomes. Odds ratios (OD) reported for stillbirths, neonatal mortality multiple births, sex ratio and preterm delivery ranged from 0.99 to 1.00.

Furthermore, epidemiologic studies alone were not able to assess the consequences of other related direct and indirect emissions and/or benefits (e.g., energy recovery, landfilling reduction, materials recovery, material consumption) associated with MSWI.

In more recent years, the potential health consequences due to atmospheric aerosols was investigated by the oxidative potential of particulate matter (PM) (OP^PM^) (West et al. 2016; Manisalidis et al. 2020; Molina et al. 2020; Canha et al. 2021). This indicator returns information about the ability of PM to generate high concentrations of reactive oxygen species (ROS) able to generate cellular damage and hence potential inflammatory effects on the respiratory apparatus of humans (Delfino et al. 2013; Li et al. 2003, 2009). Oxidative stress induced by PM is also influenced by its composition that cannot be explained by a single parameter. For this reason, the redox properties of PM referred to as OP^PM^ is considered as a promising metric for assessing health consequences caused by aerosol. Different a-cellular methods were currently proposed for assessing the OP^PM^ as e dithiothreitol (DTT) assay and the 2′,7′-dichlorofluorescin (DCFH) assay. Furthermore, many authors also investigated the correlation between the PM chemical species and the oxidative potential. Concerning the urban area of Milan, in northern Italy, Perrone et al. (2016) detected a Spearman correlation of 0.65 and 0.67 for As and Zn, respectively, contained in the sampled PM. In another study referred to the Po valley in northern Italy, Pietrogrande et al. (2024) detected a Pearson coefficient among the OP and the Cr, Zn, Cu and total metals in the sampled PM ranging from 0.66 up to 0.90.

Potential consequence can be also assessed by specific indicators in LCA studies concerning human toxicity and human health (Di Maria et al. 2020; Fernandez-Nava et al. 2014; Huang et al. 2024; Mulya et al. 2022; Yi et al. 2011; Zhang et al. 2024) even if no specific health outcomes can be detected neither quantified. In comparing the environmental impact of MSWI and landfilling Liu et al. (2021) reported global warming, freshwater toxicity potential and human toxicity potential of 6.43 kg CO_2_/tonne and 5.8 × 10^2^ kg CO_2eq_/tonne, of 1.25 × 10^−2^ kg DCBeq/tonne and 9.95 × 10^−6^ kg DCBeq/tonne and of 6.07 kg DCBeq/tonne and 0.12 kg DCBeq/tonne, respectively. In another study, Guo et al. (2018) reported greenhouse gas emission and human toxicity impact for waste incineration in Cina of 210 kg CO_2eq_/tonne and 5.60 kg DCBeq/tonne, respectively. In any case, the LCA approach shows also some limitations for a proper inclusion in the exploited models of specific aspects and features of the population and of the area of the context analysed.

Based on the previous description, each of the above methods shows advantages and limitations for the assessment of the potential impact on human health. For this reason, the present study aims to integrate OP^PM^, LCA and epidemiologic surveys for assessing the effects that MSWI can have on human health using as specific context the Italian one.

Data for LCA analysis were hence retrieved from the Italian MSWI system. Epidemiologic and OP^PM^ were determined based on literature review mainly focused on the Italian and EU areas. The ECAS and IARC database were also used for the association among the specific pollutants and the related target organs as follows: respiratory (lungs, trachea, bronchus, nose); dermal (skin); digestive (stomach, liver); urinary (kidney, bladder); hematopoietic (leukaemia, lymphoma). Finally, for consistency aims represented by legislation, technological and economic levels, the data and the literature review exploited for the present study were mainly referred to the EU area.

## Materials and methods

In the following paragraphs were reported the main methodological approaches and literature source exploited for the LCA analysis, the epidemiologic and OP^PM^ surveys. The LCA analysis was performed using as case study the Italian MSWI system. Similarly, nine out 23 epidemiologic studies concerning the EU area were referred to Italian MSWI facilities (grey dots in Fig. 2).

### The Italian MSWI system

Data concerning the operation and performances of the Italian MSWI facilities were retrieved based on official documents released by legal authorities; official reports; technical documents; scientific literature; and direct observations (Di Maria et al. 2021). From these documents, detailed information about the waste treated (i.e. municipal), the technologies adopted, the pollutant emitted, the amounts and types of energies recovered (i.e. El = only electricity; CHP = combined heat and power), the amount of waste produced including their fate, were retrieved.

Based on the amount of waste treatable on yearly basis (Di Maria et al. 2018a), the MSWI facilities were divided into three main groups: small size < 80,000 tonnes/year; medium size > 80,000 tonnes/year < 200,000 tonnes/year; large size > 200,000 tonnes year. Nowadays, about 5.3 million tonnes of municipal waste have been incinerated in about 37 moving grate MSWI, 9 of which were involved in the epidemiologic investigations. Eight of these latter were in the Emilia Romagna region, northern Italy, treating a total of about 1 Mtonne/year and one in the Lazio region, central Italy, treating about 350,000 tonnes/year. The flue gas treatment system implemented in the MSWI were in line with the ones imposed by the latest EU legislation: post combustion volumes able to maintain flue gas at temperatures ≥ 850 °C at least for 2 s; both non-catalytic and catalytic reductions of NO_*x*_; dry and wet scrubbing systems for acid compound removal; activated carbon for metals and fabric filters for dust removal.

#### LCA

According to ISO 14040 (2006), ISO 14044 (2018), ISO 14042 (2000) and the ILCD Handbook (EC, 2010) guidelines, the impact on both the environment and human health was investigated by an LCA approach. Calculations were performed by SimaPro 8.5.2 (Goedkoop et al. 2016).

### Goal, scope and functional unit

The LCA goal was the assessment of the consequences on human health and on the following main environmental aspects: global warming; freshwater ecotoxicity; particulate matter (PM) and resource depletion. The present study was carried out for research purposes, but the results can also support decision making, plant managers and other researchers.

**Fig. 3:**
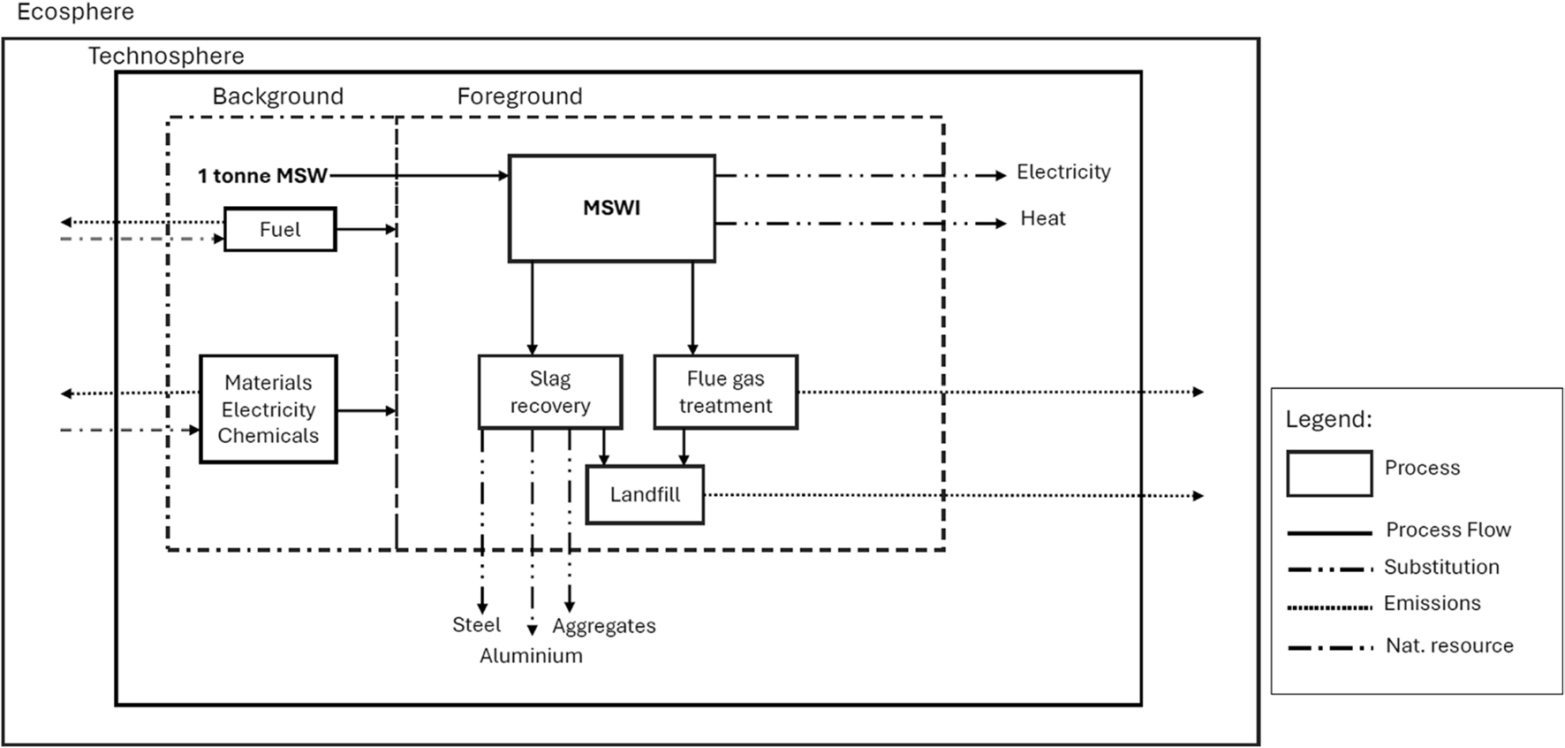
Boundaries of the system

Foregrounds were represented by energy and materials recoverable from MSWI and the related emissions due to the process including the recovery/disposal of the resulting waste. The amounts of fly ashes and slags moved to recovery, referred to as the single tonne of MSWI, are reported in S1. In the same S1 were also reported the amount of energy recoverable both electricity and heat. The main materials recovered from slags were aluminium, steel and aggregates (S1). In S2 were reported the outputs and the energy consumptions requested for the slag treatment. Backgrounds were represented by auxiliary fuels, chemicals and materials necessary for the operation of the MSWI facilities, of the landfill and of the slag treatment (S2, S3, S4). Since the foreground was not able to modify the backgrounds, the life cycle inventory was attributional. System boundaries (Fig. 3) were expanded for accounting for the transformation operated on the inlet waste by the process (*i.e.* multifunctionality). The function of the system investigated was the treatment of municipal solid waste by incineration whereas the reference flow was 1 tonne of MSWI.

### Impact indicators and assessment method

The impact was assessed using mid and end-point impact indicators. The assessment of the environmental consequences was analysed by considering the UN sustainable developing goals most affected by waste management and MSWI: goal no. 6 “Clean water and sanitation”; goal no. 12 “Responsible production and consumption”; goal no. 13 “Climate action”. For this, the following midpoint indicators were selected: global warming (GWP) (kg CO_2eq_); PM (kg PM_2.5 eq_); freshwater ecotoxicity (FWec) (CTUe); resource depletion (RD) (kg Sb_eq_). The impact on human health was assessed by the human toxicity non-cancer (HTnc) (CTUh, human toxicity cancer (HTc) (CTUh) and by the human health (HH) (DALY). The latter was an endpoint whereas the former were midpoint indicators.

According to the EC (2012), recommending the LCA impact assessment methods, the characterization for midpoint indicators was performed by the ILCD 2011 + assessment method whereas for the endpoint indicator, the IMPACT 2000 + was adopted (Jolliet et al. 2005).

### Epidemiologic survey

The main criterion for the selection of the epidemiologic studies concerning the MSWI was their relevance for the EU area. In fact, in the EU, there is homogeneity of technologies adopted, of the composition of the waste treated and on the legal limits on the concentration of pollutants. This is a consequence of quite similar economic conditions but also of a long history of the EU environmental policy that has been enforced in the main member states, since more than 40 years ago, making the results of the selected epidemiologic studies substantially comparable.

The survey was performed on the PubMed database considering the scientific studies published from 1988 to 2019. The following keywords were used: urban waste incinerators; epidemiologic study; health outcomes; carcinogen and non-carcinogen effects. From the survey were selected 23 studies differently distributed among the main EU member states: nine for Italy (Ancona et al. 2013; Candela et al. 2013; Fonte et al. 2017; Golini et al. 2014; Minichilli et al. 2016; Ranzi et al. 2011; Vinceti et al. 2009, 2018; Zambon et al. 2007), seven for France (Cordier et al. 2004; 2009; Floret et al. 2003, 2004; Goria et al. 2009; Viel et al. 2000, 2008), five for the UK (Elliott et al. 1996; Dummer et al. 2003; Ghosh et al. 2019; Lloyd et al. 1988; Williams et al. 1992) and two for Sweden (Jannson et al., 1989; Rydhstroem 1998).

Of these, 12 were descriptive/geographic studies, 7 were case–control and 5 were cohort studies. One investigation (Fonte et al. 2017) included both a case–control and a cohort study.

The investigated health outcomes included mortality, hospital admission, birth outcomes, congenital malformations, cancer (i.e. 27) and non-cancer (i.e. 43) diseases.

The nine facilities interested by the epidemiologic studies selected are highlighted in Fig. 2 (Table 1).

**Table 1:** Number of epidemiologic studies for EU member states and UK selected from the survey

EU MS	Number of studies	Years	Health outcomes investigated	
			Cancer disease	Non-cancer disease
Italy	9	2007–2018	11	24
France	7	2000–2009	9	6
UK	5	1988–2019	7	11
Sweden	2	1989–1998	0	2

### OP^PM^ survey

The survey of the OP^PM^ studies was performed focusing the attention on those reporting information on the correlation among the OP^PM^ values and the main pollutant affecting human health emitted by MSWI. Such pollutants, mainly heavy metals, corresponded to those reported in the chemical service abstract (CAS) number and international agency research on cancer (IARC) classification group (Table 2). These were also those exploited by the LCA impact assessment methodologies for the characterization of the HTc and HTnc indicators. The level of correlation was quantified by the Spearman and/or Pearson coefficients. For these specific investigations, good levels of correlation can be considered for Spearman and/or Pearson coefficient ≥ 0.6.

**Table 2:** Heavy metals from MSWI, influence on HTnc and HTc characterization, CAS and IARC classification, target organs and disease, OP assay and value (*r*) of the correlation (Spearman or Pearson)

Pollutant	Environmental media	HTnc/HTc	CAS number	IARC group	Toxicity and target organs	Carcinogenicity and target organs	OP assay/r	Reference
Sb	Air/water	Yes/no	7440–36-0	-	Respiratory Cardiovascular Digestive Haematologic	-	n.a	
As	Air/water	Yes/yes	7440–38-2	1	Respiratory Cardiovascular Nervous Development Dermal	Respiratory Dermal Urinary	DCFH/0.65^a^	Perrone et al. (2016)
Ba	Air/water	Yes/no	7440–39-3	-	Respiratory Urinary	-	AA/0.67^b^ DTT/0.62^b^	Perrone et al. (2019) Pietrogrande et al. (2018a)
Benzene	Air	Yes/yes	71–43-2	1	Immune Haematologic	Hematopoietic	n.a	
Cd	Air	Yes/yes	7440–43-9	1	Respiratory Urinary	Respiratory	DTT/0.56^b^ GSH/0.53^a^	Pietrogrande et al. (2018a, b) Szigeti et al. (2016)
CrVI	Air/water	Yes/yes	118,540–29-99	1	Respiratory Digestive Haematologic Reproductive	Respiratory	AA/0.50^b^ DTT/0.95^b^	Pietrogrande et al. (2018a, b) Visentin et al. (2016)
Cu	Air	Yes/no	744–50-8	3	Respiratory Digestive	-	AA/0.88^b^ AA/0.87^a^	Pietrogrande et al. (2018b) Szigeti et al. (2016)
Pb	Air/water	Yes/no	7439–92-1	2A	Nervous Haematopoietic Immune Urinary Cardiovascular Reproductive Development	Digestive	DTT/0.68^b^	Pietrogrande et al. (2018b)
Hg	Air	Yes/yes	7439–97-6	3	Nervous Urinary Digestive Development	-	n.a	
MoO_3_	Air	Yes/no	1313–27-5	2B	Respiratory Urinary	Respiratory	GSH/0.50^a^	Szigeti et al. (2016)
Ni	Air/water	Yes/yes	7440–02-0	1	Respiratory Haematopoietic Immune Development Skin	Respiratory	AA/0.74^b^ AA/0.59^a^	Pietrogrande et al. (2018b) Szigeti et al. (2016)
V_2_O_5_	Air	Yes/no	1314–62-1	2B	Respiratory Eyes Digestive Haematologic Development	Respiratory	AA/0.61^b^ DTT/0.77^b^	Pietrogrande et al. (2018b) Perrone et al. (2019)
Zn	Air/water	Yes/no	7440–66-6	-	Respiratory Digestive Haematologic Development	-	DCHF/0.77^a^ DTT/0.88^b^	Perrone et al. (2016) Visentin et al. (2016)

^a^Spearman

^b^Pearson

The OP^PM^ quantification can be performed by two main assay typologies: acellular and cellular. Among the acellular ones, the most diffused are the dithiothreitol (DTT) consumption rate (nmol/min*m^3^) of PM (Cho et al. 2005); the antioxidant depletion assay by the detection of ascorbic acid (AA) and glutathione (GSH) on synthetic respiratory tract (Ayres et al. 2008; Mudway et al. 2001); the plasmid scission (Donaldson et al. 1997); the DCFH probe and the CM-H2 DCF based on the oxidation of the (5- (and-6) chloromethyl-2′, 7′-dichlorodihydro-fluorescein diacetate) probe. Among the main cellular assays, there are the cell culture and particle treatment using the NCl-H292 human cell and the mRNA by RT-qPCR. According to Hedayat et al. (2015), acellular assays were largely exploited in literature for assessing the OP^PM^ of the aerosols due to traffic and the one present in urban areas.

## Results

### Impacts on the environment

**Fig. 4:**
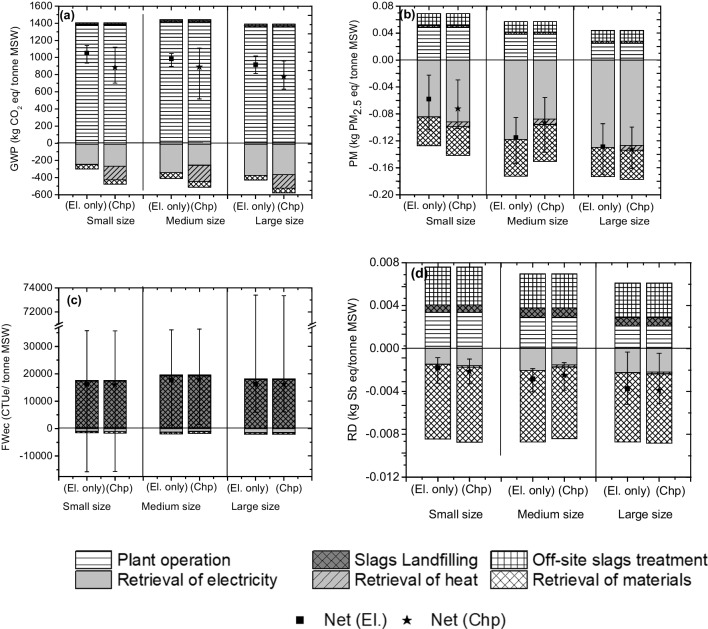
Characterization and associated uncertainty of global warming potential (GWP) (**a**), particulate matter (**b**), freshwater ecotoxicity (FWec) (**c**), resource depletion (RD) (**d**)

Larger facilities are characterized by higher energy efficiency compared to lower size. This is the main reason leading to the lower impact detected for the former compared to the latter (Fig. 4). Said this, the Italian MSWI system is characterized by net positive values of GWP, ranging from about 800 kg CO_2eq_/tonne MSW to about 1100 kg CO_2eq_/tonne MSWI (Fig. 4a). Avoided CO_2eq_ emissions due to material recovery from slag resulted negligible if compared to the one avoided for the replacement of energy from the Italian energy mix.

An important role of slag recovery was detected for the kg PM_2.5 eq_/tonne MSWI (Fig. 4b). In fact, the recovery of slags gives an important contribution to the negative values (*i.e.* avoided emissions) for this impact indicator ranging from about − 0.04 kg PM_2.5 eq_/tonne MSWI to about − 0.13 kg PM_2.5 eq_/tonne MSWI. Very similar average values of about 15,000 CTUe/tonne MSWI, concerning FWec, were detected for all the facility sizes (Fig. 4c). In addition, these values were also characterized by a high uncertainty.

Similarly, quite a constant value for all the sizes of the facilities of − 07E − 3 kg Sb_eq_/tonne MSWI was also detected for the RD (Fig. 4d). In this case, the larger contribution to RD was represented by the material recovery from the slag treatment.

### Impact on human health

**Fig. 5:**
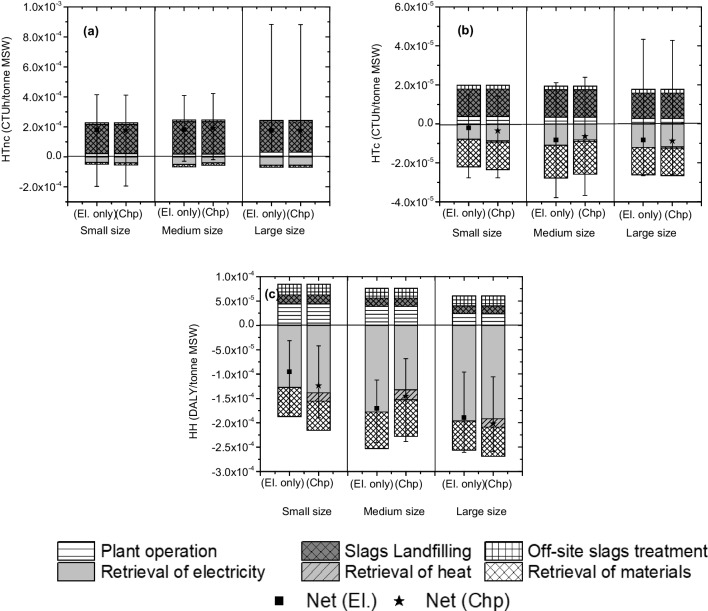
Characterization and related uncertainty of human toxicity non-cancer (HTnc) (**a**), human toxicity cancer (HTc) (**b**) and human health (HH) (**c**)

The characterization of HTnc (CTUh/tonne MSWI) (Fig. 5a) shows very similar values per each facility size with negative emissions (*i.e.* avoided) mainly due to electrical energy recovery. The contribution of the emission from incineration and slag treatment resulted negligible. Finally, the small-size facilities were characterized by the higher uncertainty.

Avoided impact due to energy and material recovery was detected also for HTc (CTUh/tonne MSWI) (Fig. 5b). Also, in this case, the uncertainty resulted quite high.

Similar results were detected for the endpoint indicator HH (DALY) (Fig. 5c) despite the uncertainty values; the avoided impacts confirmed all the facility sizes.

### Evidence of epidemiologic studies

The epidemiologic studies highlighted that, in most of the cases analysed, no association was detected for birth health outcomes and for those related to the first year of life. An exception was detected in the study of Candela (2013) in which an association among MSWI and one out three preterm births and one out four twins’ births was detected.

Similarly, in Cordier et al. (2014) and Dummer et al. (2003), few positive associations were detected between the presence of a waste incineration facility and congenital malformations (*e.g.* urinary, cardiac, facial). Furthermore, in none of the above studies was demonstrated the presence of causal correlation. In other studies, performed by Elliott et al. (1996), Goria et al. (2009) and Ranzi et al. (2011), some correlations were reported with cancer diseases. More in detail, Elliot et al. (1996) found a strong correlation between liver cancer and level of deprivation of population living within 3 km from incineration; Goria et al. (2009) found the coefficient of Poisson regression ranging from 0.687 to 1.159 for breast cancer in women and from 0.440 to 1.119 for live cancer in men related to index of exposure. Nonlinear regression was detected for male lung cancer; Ranzi et al. (2011) detected a RR vs the reference category for a cohort of residents in the area of incinerators, for all cancers due to heavy metal exposition, ranging from 0.87 to 1.00 for men and from 0.90 to 1.00 for women.

Based on hospital admission and mortality, Minichilli et al. (2016) and Fonte et al. (2017) reported some association related to non-cancer disease. Opposite results concerning non-cancer diseases were obtained by the study of Ranzi et al. (2011) and Golini et al. (2014).

Analysing hospital admission and mortality, Ancona et al. (2013) detected some association with the presence of the incineration facility and respiratory diseases. Such association was not detected in the previous studies.

Based on the studies of Ranzi et al. (2011), Ancona et al. (2013), Golini et al. (2014) and Minichilli et al. (2016), no accordance of results was observed concerning chronic obstructive pulmonary diseases whereas Minichilli et al. (2016) reported no association with digestive diseases. The last authors also reported an association with urinary diseases but not with mortality. In general, no accordance was detected concerning cardiovascular diseases.

Furthermore, the absence of a definitive demonstration of causal relationship among the different diseases and the MSWI was mainly attributable to some flaws and lacks in the methodological approach disregarding confounding factors and social conditions but also adequate analysis of the technologies adopted for the MSWI process (Lloyd, 1988).

### OP^PM^ correlations

Zinc, copper and chromium(VI) resulted the pollutants characterized by a higher correlation, > 0.8, with the OP^PM^ (Table 2) (Perrone et al. 2016; Pietrogrande et al. 2018a; Visentin et al. 2016). Zinc and Cr(IV) were toxic for the respiratory and digestive apparatus, whereas Cu was toxic for the respiratory apparatus. Carcinogenicity for the respiratory apparatus was associated (Table 2) to only Cr(VI). Correlation ≥ 0.6 but ≤ 0.8 was detected between the OP^PM^ and arsenic, barium, lead and vanadium (Perrone et al. 2016, 2019; Pietrogrande et al. 2018a, b). Arsenic resulted toxic for the respiratory, cardiovascular and nervous systems whereas resulted carcinogenic for respiratory, dermal and urinary systems. Barium was characterized by toxicity for the respiratory and urinary apparatus whereas lead resulted to have toxic effect for the nervous, hematopoietic and immune system and carcinogenic for the digestive system. Finally, vanadium resulted carcinogenic for the respiratory apparatus and toxic for the respiratory, eyes and digestive apparatus. Correlations < 0.6 were detected for cadmium and molybdenum (trioxide) both resulting toxic and carcinogenic for the respiratory system (Pietrogrande et al. 2019; Szigeti et al. 2016).

## Discussion

### Environmental impact

Values of the GWP related to MSWI were controversial in literature. In fact, Boesch et al. (2014) and Rigamonti et al. (2013) reported positive values of about 127 kg CO_2eq_/tonne MSWI to about 425 kg CO_2eq_/tonne MSWI, respectively. On the contrary, negative GWP up to − 109 kg CO_2eq_/tonne MSWI were found by Beylot and Villeneuve (2013). The same authors also detected PM impacts ranging from − 0.4 to 0.35 kg PM_10eq_/tonne MSWI, indicating a direct correlation between the increase in energy efficiency and the impact reduction.

Beylot et al. (2018) and Fruergaard and Astrup (2011) reported avoided FWec impact was largely influenced by the recovery of materials and energy. Concerning RD (kg Sb_eq_/tonne MSWI), opposite findings were reported by Morselli et al. (2008) and by Yay (2015). For the former, MSWI was able to lead to negative (*i.e.* avoided) values whereas for the latter, MSWI was charged of positive values (*i.e.* impact). All these highlight the influence that local conditions (*e.g.* energy mix replaced, industrial process avoided, amount of material recycled, MSWI technologies) and the related assumptions in building the inventories can have on LCA results.

### Health impact

Based on LCA, avoided human toxicity due to airborne emission was reported by Turconi et al. (2011), but opposite findings were reported by Fruergaard and Astrup (2011). Both studies reported also avoided impact due to soil and water contamination.

Positive impact on human health (DALY) due to MSWI was reported also by Morselli et al. (2008).

Again, for environmental impact, local conditions and the associated assumption in building inventories are able to be more representative of the specific context and can influence the final results of the LCA studies. As expected, the findings reported in epidemiologic studies were coherent with the CAS and IARC databases (Table 2). Furthermore, the presence of several methodological flaws led to the impossibility of demonstrating a direct causal correlation between the specific disease and the MSWI emission.

**Fig. 6:**
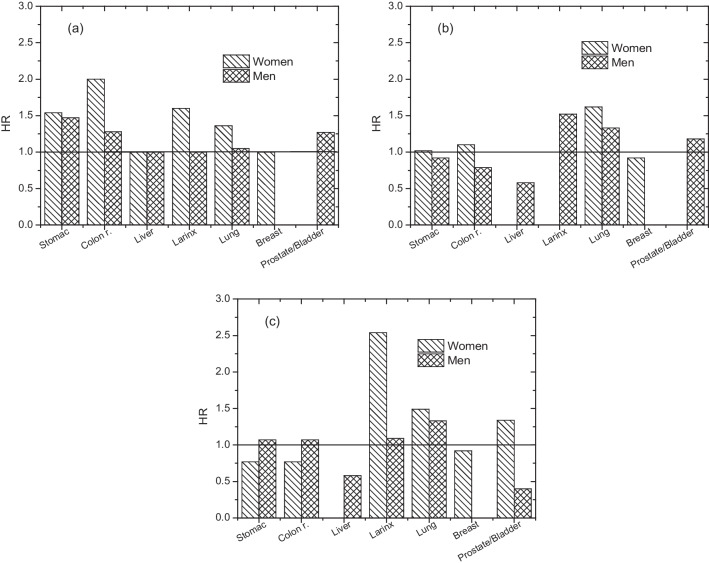
Max values of hazard ratios (HR) reported in literature for cancer incidence in populations exposed to MSWI facilities. (**a** Ranzi et al. 2011; **b** Ancona et al. 2013; **c** Minichilli et al. 2016)

More in detail, LCA gives an impression of the potential impacts or benefits, very useful for example in forecasting studies, but without returning any quantification of final health outcomes. Concerning the main findings reported in the epidemiologic survey (Fig. 6), there are no definitive agreements on the effective higher incidences of specific diseases for populations exposed to incineration facilities (*i.e.* HR ≤ 1). On the contrary, in some cases, a lower incidence of some cancer was detected for exposed populations compared to not exposed ones (Fig. 6b,c). These results indicate that epidemiologic studies can be potentially able to quantify health outcomes, but the complexity of the phenomena investigated introduces too many variables and confounding factors, making it very difficult to always achieve a definitive tether between the specific source of the emission and the related health outcome. Finally, epidemiologic studies were generally exploited for retrospective investigations.

More direct information on possible health impact due to MSWI in the analysed context can be found in biological biomonitoring. Ruggieri et al. (2019) report on a spatial and temporal trend of exposure to 18 heavy metals in a cohort of subject exposed to an MSWI facility located in northern-west Italy. The period of investigation included the year before and the year after the MSWI facility activation. Main findings indicate that there was a generalized decrease in the heavy metal concentration in urine samples after the MSWI activation.

For the same MSWI facility, Iamicelia et al. (2020) found a lower concentration of some polycyclic aromatic hydrocarbon metabolites in the urine samples of those monitored subjects living close to the plant. Some explanation to these positive effects can be found in the reduction of the emissions caused by the heat recovered by the MSWI process and exploited for the heating of civil and public buildings. In this way, the emissions due to fossil- and/or biomass-fuelled heat generators were avoided decreasing the emission of such pollutants in the atmosphere.

The results reported by Ruggieri et al. (2019) and Iamicelia et al. (2020) were in line with those reported by previous studies (Wultsch et al. 2011) in which no differences were detected in the concentration of urinary heavy metals and lymphocyte genotoxic effect, between exposed and not exposed subjects to MSWI.

In this context, the correlation of OP^PM^ with specific heavy metals was coherent with the findings above reported mainly in confirming their potential toxic effects on the respiratory apparatus. In fact, based on Table 2, the heavy metals showing higher correlation with the values of the OP^PM^ were CrVI, Cu and Zn. Both CAS and IARC indicated the toxic effects of these heavy metals on the respiratory tract. Potential carcinogenic effect on the respiratory apparatus was reported only for Cr(VI). Possible correlation with urinary disease reported by Minichilli et al. (2016) can be found; some match the correlation of the value of OP^PM^ with the presence of Ba for toxic effects and As for cancer effects (Tabel 2). The Pearson/Spearman correlation reported among Ba, As and OP^PM^ was not noticeably lower than the one reported for Cr(VI), Cu and Zn.

For the other potential diseases affecting other target organs (*e.g.* digestive and nervous apparatus), the low values of the correlation with the specific heavy metal and the OP^PM^ (*i.e.* < 0.6) were in line with the findings of the epidemiologic studies. Of course, the presence of aerosol and the related pollutants in the urban areas has manifold sources among which MSWI represents a marginal one. The high correlation detected among the OP^PM^ and some pollutants can be another important information to be associated with those returned by LCA and epidemiologic studies for better understanding of the potential nexus with specific health outcomes.

## Conclusions

The investigation of possible consequences on human health due to the emissions generated by both anthropogenic activities and natural phenomena is a critical but necessary aspect in modern society for giving the necessary information to decision makers but also to the large public. This is a complex operation, accounting for several aspects and confounding factors, requiring multidisciplinary approaches and quite long investigation period. In modern and industrialized areas, this complexity rises when the goal is to focus the analysis on a specific facility or process. In this case, the presence of many different confounding factors, not always easy to account, can lead to bias or not exhaustive results making the whole investigation and related results not able to deliver the expected information. This was also confirmed by the main findings reported in the present study, related to populations exposed to waste incinerators, highlighting risk ratio, hazard ratio and odds ratio values, referred to the same health outcomes that in many cases were in contrast each other and often also < 1.

For this reason, the availability of affordable and less complex approaches able to return useful information and/or indication, both retrospective and predictive, in a lower timeframe is of interest in this sector.

The adoption of simplified investigations methodologies based on LCA but also on the measurement of the oxidative potential of atmospheric aerosol can return information that, based on the findings reported in the present study, resulted in line with those returned by epidemiologic analysis. Hence, even if more research results are necessary in the future for better understanding of these phenomena, the present study confirmed the importance and the necessity of multidisciplinary and integrated approaches for supporting knowledge and decision makers on this critical but very important sector.

## Supplementary Information

Below is the link to the electronic supplementary material.

Supplementary file1 (DOCX 25 KB)

Supplementary file2 (DOCX 23 KB)

Supplementary file3 (DOCX 56 KB)

Supplementary file4 (DOCX 55 KB)

## Data Availability

All the data related to the manuscript have been reported within the manuscript and in the Supplementary material S1, S2, S3 and S4.
