# Supplementary material for: Supporting the investigation of health outcomes due to airborne emission by different approaches: current evidence for the waste incineration sector
Source: Environ Sci Pollut Res Int. 2024 Sep 24;31(48):58527–40. doi: 10.1007/s11356-024-34989-x (PMC11467001; doi:10.1007/s11356-024-34989-x)
Supplement: Supplementary file 1 — Supplementary file1 (DOCX 25 KB) [file 11356_2024_34989_MOESM1_ESM.docx]

**S1.** Average values and standard deviation of the amount of energy and materials recovered from the Italian MSWI facilities (CiAl, 2010; ISPRA, 2018).

|  | Small size | Medium size | Large size | |  |
| --- | --- | --- | --- | --- | --- |
|  | (Avg./std. dev.) | (Avg./std. dev.) | (Avg./std. dev.) | | |
| *Energy recovery*  *(kWh/tonne MSWI)* |  |  |  |  |  |
| Electricity (El) | 496/138 | 687/66 | 762/141 |  |  |
| Electricity (CHP) | 448/185 | 514/92 | 745/75 |  |  |
| Heat (CHP) | 1102/243 | 798/755 | 732/367 |  |  |
| *Ash and Slags (tonne/tonne MSW)* |  |  |  |  |  |
| Ash to disposal ^a^ | 3.6E-02/2E-2 | 4.5E-02/1.4E-2 | 4.0E-02/7E-3 |  |  |
| Slags to off-site recovery | 1.9E-01/5E-2 | 1.7E-01/3E-2 | 1.8E-01/2E-2 |  |  |
| Slags to disposal | 2.2E-02/6E-3 | 2.0E-02/4E-3 | 2.0E-02/2.5E-3 |  |  |
| On-site iron scraps recovery | 1.3E-03/5E-4 | 1.1E-02/5E-3 | 3.8E-03/2E-3 |  |  |

Legend:

^a^ expressed as the sum of hazardous bottom and fly ashes and flue gas cleaning waste and sludge.
