# Supplementary material for: Supporting the investigation of health outcomes due to airborne emission by different approaches: current evidence for the waste incineration sector
Source: Environ Sci Pollut Res Int. 2024 Sep 24;31(48):58527–40. doi: 10.1007/s11356-024-34989-x (PMC11467001; doi:10.1007/s11356-024-34989-x)
Supplement: Supplementary file 2 — Supplementary file2 (DOCX 23 KB) [file 11356_2024_34989_MOESM2_ESM.docx]

# S2. Material outputs and energy consumption for the off-site treatment of 1 tonne of slags from MSWI.

| **Parameter** |  | **Unit** |
| --- | --- | --- |
| *Outputs* |  |  |
| Aluminum scrap | 13.3 | kg |
| Iron scrap | 78.1 | kg |
| Inert material | 751 | kg |
| Water | 70 | kg |
| Waste to landfill | 87.6 | kg |
| *Energy consumption* |  |  |
| Electricity | 4 | kWh |
