# Supplementary material for: Supporting the investigation of health outcomes due to airborne emission by different approaches: current evidence for the waste incineration sector
Source: Environ Sci Pollut Res Int. 2024 Sep 24;31(48):58527–40. doi: 10.1007/s11356-024-34989-x (PMC11467001; doi:10.1007/s11356-024-34989-x)
Supplement: Supplementary file 3 — Supplementary file3 (DOCX 56 KB) [file 11356_2024_34989_MOESM3_ESM.docx]

**S3**

Inventory for the incineration of 1 tonne of MSWI. Geometric mean (μ_g_) and variance(σ^2^) were referred to a Lognormal distribution.

| **Parameter** |  | **Small size (µg/****σ^2^)** | **Medium size (µg/σ^2^)** | **Large size (µg/σ^2^)** | **Unit** |
| --- | --- | --- | --- | --- | --- |
| *Materials/fuels* |  |  |  |  |  |
| Ammonia, liquid |  | 1.16E-03/5.13 | 2.71E-03/5.13 | 2.13E-03/5.13 | kg |
| Chemical, inorganic | | 2.18E-04/2.86 | 2.18E-04/2.86 | 5.68E-04/2.86 | kg |
| Chemical, organic |  | 1.28E-05/10.67 | 1.28E-05/10.67 | 1.28E-05/10.67 | kg |
| Chromium oxide, flakes |  | 8.94E-07/7.46 | 8.94E-07/7.46 | 8.94E-07/7.46 | kg |
| Heat, district or industrial, natural gas | | 1.11E-01/5.13 | 1.31E-01/5.13 | 6.69E-02/5.13 | MJ |
| Hydrochloric acid | | 4.34E-04/2.86 | 9.86E-07/2.86 | 1.52E-04/2.86 | kg |
| Iron (III) chloride | | 0.00E+00/2.65 | 3.94E-05/2.65 | 2.42E-04/2.65 | kg |
| Municipal waste incineration facility | | 2.50E-10/ | 2.50E-10/ | 2.50E-10/ | p |
| Quicklime, milled, packed | | 3.55E-03/3.53 | 6.80E-03/3.53 | 5.48E-03/3.53 | kg |
| Sodium hydroxide | | 1.04E-03/2.52 | 3.89E-04/2.52 | 2.80E-04/2.52 | kg |
| Titanium dioxide |  | 4.38E-05/7.46 | 4.38E-05/7.46 | 4.38E-05/7.46 | kg |
| Water, decarbonised, at user |  | 1.39E+00/2.65 | 1.39E+00/2.65 | 1.39E+00/2.65 | kg |
| On-site sorting and pressing of iron scrap | | 1.35E-03 | 1.08E-02 | 3.85E-03 | kg |
| Urea formaldehyde resin | | 3.67E-03 | 4.93E-04 | 5.20E-04 | kg |
| Activated carbon | | 1.42E-03 | 7.01E-04 | 4.50E-04 | kg |
| Sulfuric acid |  | 3.29E-04 | 1.82E-04 | 2.50E-04 | kg |
| Sodium bicarbonate | | 1.06E-02 | 1.08E-02 | 5.52E-03 | kg |
| Diesel |  | 8.38E-04 | 1.07E-03 | 2.06E-04 | kg |
|  |  |  |  |  |  |
| *Emissions to air* |  |  |  |  |  |
| Aluminium | low. pop. | 5.69E-09/3.69 | 5.69E-09/3.69 | 5.69E-09/3.69 | kg |
| Ammonia, IT | low. pop. | 8.29E-06/5.47 | 4.84E-06/5.47 | 5.64E-06/5.47 | kg |
| Antimony | low. pop. | 2.93E-08/8.29 | 2.14E-08/8.29 | 6.03E-09/8.29 | kg |
| Arsenic | low. pop. | 3.19E-08/11.90 | 2.33E-08/11.90 | 6.57E-09/11.90 | kg |
| Barium | low. pop. | 1.45E-07/6.86 | 1.45E-07/6.86 | 1.45E-07/6.86 | kg |
| Benzene | low. pop. | 4.33E-08/1.89 | 5.63E-08/1.89 | 5.39E-08/1.89 | kg |
| Bromine | low. pop. | 6.46E-08/7.02 | 6.46E-08/7.02 | 6.46E-08/7.02 | kg |
| Cadmium | low. pop. | 1.03E-09/9.99 | 1.65E-10/9.99 | 3.60E-10/9.99 | kg |
| Calcium | low. pop. | 9.04E-05/3.03 | 9.04E-05/3.03 | 9.04E-05/3.03 | kg |
| Carbon dioxide, biogenic | low. pop. | 7.52E-01/1.43 | 7.52E-01/1.43 | 7.52E-01/1.43 | kg |
| Carbon dioxide, fossil | low. pop. | 4.78E-01/1.43 | 4.78E-01/1.43 | 4.78E-01/1.43 | kg |
| Carbon monoxide, biogenic | low. pop. | 1.55E-05/6.30 | 1.63E-05/6.30 | 3.10E-05/6.30 | kg |
| Carbon monoxide, fossil | low. pop. | 9.85E-06/6.30 | 1.03E-05/6.30 | 1.97E-05/6.30 | kg |
| Chromium | low. pop. | 1.19E-08/7.02 | 8.68E-09/7.02 | 2.45E-09/7.02 | kg |
| Cobalt | low. pop. | 1.17E-08/12.04 | 8.56E-09/12.04 | 2.42E-09/12.04 | kg |
| Copper | low. pop. | 1.66E-08/5.38 | 1.21E-08/5.38 | 3.42E-09/5.38 | kg |
| Cyanide | low. pop. | 1.03E-05/9.62 | 1.03E-05/9.62 | 1.03E-05/9.62 | kg |
| Dinitrogen monoxide | low. pop. | 4.78E-05/9.62 | 4.78E-05/9.62 | 4.78E-05/9.62 | kg |
| Dioxin | low. pop. | 1.69E-14/3.78 | 1.96E-14/3.78 | 1.72E-14/3.78 | kg |
| Heat, waste | low. pop. | 9.58E+00/1.61 | 9.58E+00/1.61 | 9.58E+00/1.61 | MJ |
| Hydrogen chloride | low. pop. | 9.72E-06/3.77 | 9.45E-06/3.77 | 9.46E-06/3.77 | kg |
| Hydrogen fluoride | low. pop. | 4.35E-07/5.99 | 4.88E-07/5.99 | 4.47E-07/5.99 | kg |
| Iodine | low. pop. | 6.89E-12/16.24 | 6.89E-12/16.24 | 6.89E-12/16.24 | kg |
| Iron | low. pop. | 1.18E-08/3.24 | 1.18E-08/3.24 | 1.18E-08/3.24 | kg |
| Lead | low. pop. | 1.34E-08/6.05 | 9.79E-09/6.05 | 2.76E-09/6.05 | kg |
| Manganese | low. pop. | 1.30E-07/6.50 | 2.01E-06/4.45 | 2.01E-06/4.45 | kg |
| Mercury | low. pop. | 3.45E-09/12.82 | 9.46E-08/6.50 | 2.67E-08/6.50 | kg |
| Magnesium | low. pop. | 2.01E-06/4.45 | 3.05E-09/12.82 | 2.17E-08/12.82 | kg |
| Methane, biogenic | low. pop. | 3.97E-07/1.89 | 5.16E-07/1.89 | 4.94E-07/1.89 | kg |
| Methane, fossil | low. pop. | 2.52E-07/1.89 | 3.28E-07/1.89 | 3.14E-07/1.89 | kg |
| Molybdenum | low. pop. | 1.34E-09/11.56 | 1.34E-09/11.56 | 1.34E-09/11.56 | kg |
| Nickel | low. pop. | 1.27E-08/7.90 | 9.23E-09/7.90 | 2.61E-09/7.90 | kg |
| Nitrogen Oxides | low. pop. | 6.25E-04/5.47 | 2.83E-04/5.47 | 2.62E-04/5.47 | kg |
| NMVOC | low. pop. | 1.97E-06/1.89 | 2.56E-06/1.89 | 2.45E-06/1.89 | kg |
| Particulates, < 2.5 um | low. pop. | 3.57E-06/3.78 | 1.90E-06/3.78 | 1.57E-06/3.78 | kg |
| Particulates, > 2.5 um, and < 10um | low. pop. | 2.05E-08/2.52 | 2.05E-08/2.52 | 2.05E-08/2.52 | kg |
| Phenol, pentachloro- | low. pop. | 1.88E-11/1.89 | 2.45E-11/1.89 | 2.35E-11/1.89 | kg |
| Phosphorus | low. pop. | 7.62E-07/5.43 | 7.62E-07/5.43 | 7.62E-07/5.43 | kg |
| Potassium | low. pop. | 1.05E-05/4.54 | 1.05E-05/4.54 | 1.05E-05/4.54 | kg |
| Selenium | low. pop. | 3.72E-09/13.76 | 3.72E-09/13.76 | 3.72E-09/13.76 | kg |
| Silicon | low. pop. | 2.49E-08/2.79 | 2.49E-08/2.79 | 2.49E-08/2.79 | kg |
| Sodium | low. pop. | 2.34E-05/3.96 | 2.34E-05/3.96 | 2.34E-05/3.96 | kg |
| Sulfur oxides, IT | low. pop. | 1.34E-05/4.80 | 1.12E-05/4.80 | 8.70E-06/4.80 | kg |
| Tin | low. pop. | 1.90E-09/7.67 | 1.38E-09/7.67 | 3.90E-10/7.67 | kg |
| Titanium | low. pop. | 1.33E-06/4.45 | 1.33E-06/4.45 | 1.33E-06/4.45 | kg |
| Toluene | low. pop. | 8.65E-08/1.89 | 1.13E-07/1.89 | 1.08E-07/1.89 | kg |
| Vanadium | low. pop. | 1.40E-08/9.67 | 1.02E-08/9.67 | 2.89E-09/9.67 | kg |
| Water/m3 | low. pop. | 1.46E-03/5.15 | 1.46E-03/5.15 | 1.46E-03/5.15 | m3 |
| Zinc | low. pop. | 1.06E-07/5.11 | 1.11E-07/5.11 | 2.50E-07/5.11 | kg |
| Thallium | low. pop. | 2.13E-08 | 3.40E-09 | 7.42E-09 | kg |
|  |  |  |  |  |  |
| *Emissions to water* |  |  |  |  |  |
| Aluminium | river | 4.11E-08/5.58 | 4.11E-08/5.58 | 4.11E-08/5.58 | kg |
| Antimony | river | 4.09E-09/7.73 | 4.09E-09/7.73 | 4.09E-09/7.73 | kg |
| Barium | river | 1.94E-09/13.46 | 1.94E-09/13.46 | 1.94E-09/13.46 | kg |
| Beryllium | river | 2.79E-08/10.11 | 2.79E-08/10.11 | 2.79E-08/10.11 | kg |
| BOD_5_ | river | 3.87E-04/1.59 | 3.87E-04/1.59 | 3.87E-04/1.59 | kg |
| Bromine | river | 4.16E-05/3.73 | 4.16E-05/3.73 | 4.16E-05/3.73 | kg |
| Cadmium | river | 4.02E-10/8.39 | 4.02E-10/8.39 | 4.02E-10/8.39 | kg |
| Calcium | river | 9.03E-05/3.02 | 9.03E-05/3.02 | 9.03E-05/3.02 | kg |
| Chloride | river | 4.00E-03/2.87 | 4.00E-03/2.87 | 4.00E-03/2.87 | kg |
| Chromium VI | river | 3.71E-09/7.37 | 3.71E-09/7.37 | 3.71E-09/7.37 | kg |
| Cobalt | river | 6.44E-12/17.98 | 6.44E-12/17.98 | 6.44E-12/17.98 | kg |
| COD | river | 3.87E-04/1.58 | 3.87E-04/1.58 | 3.87E-04/1.58 | kg |
| Copper | river | 4.22E-08/6.10 | 4.22E-08/6.10 | 4.22E-08/6.10 | kg |
| DOC | river | 1.69E-04/1.58 | 1.69E-04/1.58 | 1.69E-04/1.58 | kg |
| Fluoride | river | 1.66E-05/4.00 | 1.66E-05/4.00 | 1.66E-05/4.00 | kg |
| Heat, waste | river | 2.55E+00/1.61 | 2.55E+00/1.61 | 2.55E+00/1.61 | MJ |
| Iodide | river | 1.24E-08/10.97 | 1.24E-08/10.97 | 1.24E-08/10.97 | kg |
| Iron | river | 3.63E-08/6.91 | 3.63E-08/6.91 | 3.63E-08/6.91 | kg |
| Lead | river | 2.06E-08/5.46 | 2.06E-08/5.46 | 2.06E-08/5.46 | kg |
| Magnesium | river | 2.50E-06/5.89 | 2.50E-06/5.89 | 2.50E-06/5.89 | kg |
| Manganese | river | 5.55E-09/11.41 | 5.55E-09/11.41 | 5.55E-09/11.41 | kg |
| Mercury | river | 3.29E-10/9.31 | 3.29E-10/9.31 | 3.29E-10/9.31 | kg |
| Molybdenum | river | 3.84E-10/6.98 | 3.84E-10/6.98 | 3.84E-10/6.98 | kg |
| Nickel | river | 1.20E-09/13.35 | 1.20E-09/13.35 | 1.20E-09/13.35 | kg |
| Nitrate | river | 3.48E-05/3.29 | 3.48E-05/3.29 | 3.48E-05/3.29 | kg |
| Phosphate | river | 3.67E-07/4.26 | 3.67E-07/4.26 | 3.67E-07/4.26 | kg |
| Potassium | river | 1.09E-05/3.15 | 1.09E-05/3.15 | 1.09E-05/3.15 | kg |
| Selenium | river | 4.32E-10/10.48 | 4.32E-10/10.48 | 4.32E-10/10.48 | kg |
| Silicon | river | 2.89E-08/4.52 | 2.89E-08/4.52 | 2.89E-08/4.52 | kg |
| Silver | river | 1.47E-09/12.30 | 1.47E-09/12.30 | 1.47E-09/12.30 | kg |
| Sodium | river | 2.30E-05/2.58 | 2.30E-05/2.58 | 2.30E-05/2.58 | kg |
| Sulfate | river | 6.17E-04/2.89 | 6.17E-04/2.89 | 6.17E-04/2.89 | kg |
| Tin | river | 5.03E-11/11.73 | 5.03E-11/11.73 | 5.03E-11/11.73 | kg |
| Titanium | river | 4.22E-07/3.68 | 4.22E-07/3.68 | 4.22E-07/3.68 | kg |
| TOC | river | 1.69E-04/1.58 | 1.69E-04/1.58 | 1.69E-04/1.58 | kg |
| Vanadium | river | 9.14E-11/11.37 | 9.14E-11/11.37 | 9.14E-11/11.37 | kg |
| Water, IT | river | 4.64E-04/7.74 | 4.64E-04/7.74 | 4.64E-04/7.74 | m3 |
| Zinc | river | 5.67E-08/4.67 | 5.67E-08/4.67 | 5.67E-08/4.67 | kg |

Legend:

low. pop.= low population
