# Supplementary material for: Supporting the investigation of health outcomes due to airborne emission by different approaches: current evidence for the waste incineration sector
Source: Environ Sci Pollut Res Int. 2024 Sep 24;31(48):58527–40. doi: 10.1007/s11356-024-34989-x (PMC11467001; doi:10.1007/s11356-024-34989-x)
Supplement: Supplementary file 4 — Supplementary file4 (DOCX 55 KB) [file 11356_2024_34989_MOESM4_ESM.docx]

**S4**

Inventory for the disposal in landfill of slags and fly ashes referred to 1 kg of MSWI. Geometric mean (µ_g_) and variance (σ^2^) were referred to a Lognormal distribution.

| **Parameter** |  | **Small size (µg/****σ^2^)** | **Medium size (µg/σ^2^)** | **Large size (µg/σ^2^)** | **Unit** |
| --- | --- | --- | --- | --- | --- |
| *Materials/fuels* |  |  |  |  |  |
| Cement, unspecified | | 1.41E-02/1.48 | 1.78E-02/1.47 | 1.59E-02/1.48 | kg |
| Process-specific burdens, residual material landfill | | 3.56E-02/1.40 | 4.51E-02/1.40 | 4.04E-02/1.40 | kg |
| Process-specific burdens, slag landfill | | 2.21E-02/1.49 | 1.99E-02/1.49 | 2.01E-02/1.49 | kg |
| Residual material landfill infrastructure | | 7.41E-11/1.40 | 9.33E-11/1.40 | 1.92E-10/1.40 | p |
| Slag landfill infrastructure | | 3.92E-11/1.49 | 3.51E-11/1.49 | 3.55E-11/1.49 | p |
|  |  |  |  |  |  |
| *Emissions to water* |  |  |  |  |  |
| Aluminium | river | 1.79E-07/5.92 | 2.01E-07/5.92 | 1.87E-07/5.92 | kg |
| Aluminium | g.w., l.t. | 1.81E-03/3.24 | 2.04E-03/3.24 | 1.90E-03/3.24 | kg |
| Arsenic | g.w., l.t. | 1.65E-07/11.56 | 1.86E-07/11.56 | 1.73E-07/11.56 | kg |
| Arsenic | river | 1.65E-07/10.51 | 1.86E-07/10.51 | 1.73E-07/10.51 | kg |
| BOD_5_ | g.w., l.t. | 3.60E-04/1.64 | 4.06E-04/1.64 | 3.78E-04/1.64 | kg |
| BOD_5_ | river | 1.18E-06/1.96 | 1.33E-06/1.96 | 1.23E-06/1.96 | kg |
| Boron | g.w., l.t. | 1.48E-06/8.29 | 1.67E-06/8.29 | 1.55E-06/8.29 | kg |
| Boron | river | 1.37E-08/11.65 | 1.54E-08/11.65 | 1.43E-08/11.65 | kg |
| Bromine | g.w., l.t. | 2.94E-06/10.24 | 3.32E-06/10.24 | 3.09E-06/10.24 | kg |
| Bromine | river | 1.78E-05/5.34 | 2.01E-05/5.34 | 1.87E-05/5.34 | kg |
| Cadmium | river | 9.78E-11/11.86 | 1.10E-10/11.86 | 1.03E-10/11.86 | kg |
| Cadmium | g.w., l.t. | 1.64E-07/221.7 | 1.84E-07/221.7 | 1.72E-07/221.7 | kg |
| Calcium | g.w., l.t. | 3.63E-03/2.85 | 4.09E-03/2.85 | 3.81E-03/2.85 | kg |
| Calcium | river | 1.57E-05/2.70 | 1.77E-05/2.70 | 1.65E-05/2.70 | kg |
| Chloride | g.w., l.t. | 3.50E-04/4.08 | 3.94E-04/4.08 | 3.67E-04/4.08 | kg |
| Chloride | river | 2.80E-04/3.55 | 3.16E-04/3.55 | 2.94E-04/3.55 | kg |
| Chromium | river | 8.65E-10/10.22 | 9.75E-10/10.22 | 9.07E-10/10.22 | kg |
| Chromium VI | g.w., l.t. | 7.10E-07/14.32 | 8.00E-07/14.32 | 7.45E-07/14.32 | kg |
| Chromium VI | river | 1.62E-07/10.52 | 1.83E-07/10.52 | 1.70E-07/10.52 | kg |
| Cobalt | river | 5.07E-11/13.24 | 5.71E-11/13.24 | 5.31E-11/13.24 | kg |
| Cobalt | g.w., l.t. | 2.95E-07/9.74 | 3.33E-07/9.74 | 3.10E-07/9.74 | kg |
| COD | river | 3.60E-06/1.94 | 4.05E-06/1.94 | 3.77E-06/1.94 | kg |
| COD | g.w., l.t. | 1.10E-03/1.64 | 1.24E-03/1.64 | 1.16E-03/1.64 | kg |
| Copper | river | 8.02E-09/7.94 | 9.04E-09/7.94 | 8.41E-09/7.94 | kg |
| Copper | g.w., l.t. | 1.78E-04/8.84 | 2.00E-04/8.84 | 1.87E-04/8.84 | kg |
| DOC | river | 1.42E-06/1.95 | 1.60E-06/1.95 | 1.49E-06/1.95 | kg |
| DOC | g.w., l.t. | 4.36E-04/1.64 | 4.91E-04/1.64 | 4.57E-04/1.64 | kg |
| Fluoride | river | 2.69E-06/4.38 | 3.03E-06/4.38 | 2.82E-06/4.38 | kg |
| Fluoride | g.w., l.t. | 7.76E-05/4.09 | 8.74E-05/4.09 | 8.13E-05/4.09 | kg |
| Heat, waste | river | 3.53E-01/1.89 | 3.97E-01/1.89 | 3.70E-01/1.89 | MJ |
| Iron | g.w., l.t. | 1.68E-03/6.78 | 1.89E-03/6.78 | 1.76E-03/6.78 | kg |
| Iron | river | 4.67E-08/7.29 | 5.26E-08/7.29 | 4.90E-08/7.29 | kg |
| Lead | river | 1.49E-09/7.48 | 1.68E-09/7.48 | 1.56E-09/7.48 | kg |
| Lead | g.w., l.t. | 6.72E-05/64.56 | 7.57E-05/64.56 | 7.05E-05/64.56 | kg |
| Magnesium | g.w., l.t. | 5.55E-04/4.24 | 6.25E-04/4.24 | 5.82E-04/4.24 | kg |
| Magnesium | river | 2.10E-06/5.48 | 2.37E-06/5.48 | 2.20E-06/5.48 | kg |
| Manganese | g.w., l.t. | 5.86E-05/21.40 | 6.60E-05/21.40 | 6.15E-05/21.40 | kg |
| Manganese | river | 3.06E-09/8.88 | 3.44E-09/8.88 | 3.21E-09/8.88 | kg |
| Mercury | river | 1.30E-10/10.66 | 1.46E-10/10.66 | 1.36E-10/10.66 | kg |
| Mercury | g.w., l.t. | 1.13E-08/40.52 | 1.27E-08/40.52 | 1.18E-08/40.52 | kg |
| Molybdenum | river | 7.64E-08/9.15 | 8.61E-08/9.15 | 8.01E-08/9.15 | kg |
| Molybdenum | g.w., l.t. | 3.97E-07/13.60 | 4.47E-07/13.60 | 4.16E-07/13.60 | kg |
| Nickel | g.w., l.t. | 1.21E-05/10.09 | 1.36E-05/10.09 | 1.27E-05/10.09 | kg |
| Nickel | river | 7.77E-09/15.88 | 8.75E-09/15.88 | 8.15E-09/15.88 | kg |
| Nitrate | river | 1.43E-06/3.25 | 1.61E-06/3.25 | 1.50E-06/3.25 | kg |
| Nitrate | g.w., l.t. | 2.72E-05/4.13 | 3.07E-05/4.13 | 2.86E-05/4.13 | kg |
| Phosphate | g.w., l.t. | 2.38E-05/79.00 | 2.68E-05/79.00 | 2.50E-05/79.00 | kg |
| Phosphate | river | 1.30E-08/4.14 | 1.46E-08/4.14 | 1.36E-08/4.14 | kg |
| Potassium | river | 1.07E-04/3.00 | 1.21E-04/3.00 | 1.12E-04/3.00 | kg |
| Potassium | g.w., l.t. | 3.92E-04/2.75 | 4.41E-04/2.75 | 4.11E-04/2.75 | kg |
| Selenium | g.w., l.t. | 5.15E-08/9.38 | 5.80E-08/9.38 | 5.40E-08/9.38 | kg |
| Selenium | river | 2.49E-08/10.23 | 2.81E-08/10.23 | 2.61E-08/10.23 | kg |
| Silicon | river | 1.21E-06/4.43 | 1.37E-06/4.43 | 1.27E-06/4.43 | kg |
| Silicon | g.w., l.t. | 4.50E-04/82.37 | 5.06E-04/82.37 | 4.71E-04/82.37 | kg |
| Sodium | g.w., l.t. | 8.77E-04/3.04 | 9.88E-04/3.04 | 9.20E-04/3.04 | kg |
| Sodium | river | 2.32E-04/3.09 | 2.61E-04/3.09 | 2.43E-04/3.09 | kg |
| Sulfate | g.w., l.t. | 9.02E-04/3.28 | 1.02E-03/3.28 | 9.46E-04/3.28 | kg |
| Sulfate | river | 3.24E-05/2.94 | 3.65E-05/2.94 | 3.39E-05/2.94 | kg |
| Tin | river | 5.53E-10/16.24 | 6.22E-10/16.24 | 5.79E-10/16.24 | kg |
| Tin | g.w., l.t. | 1.24E-05/18.94 | 1.39E-05/18.94 | 1.30E-05/18.94 | kg |
| TOC | river | 1.42E-06/1.95 | 1.60E-06/1.95 | 1.49E-06/1.95 | kg |
| TOC, | g.w., l.t. | 4.36E-04/1.64 | 4.91E-04/1.64 | 4.57E-04/1.64 | kg |
| Vanadium | river | 1.42E-09/10.95 | 1.60E-09/10.95 | 1.49E-09/10.95 | kg |
| Vanadium | g.w., l.t. | 3.63E-07/8.14 | 4.09E-07/8.14 | 3.81E-07/8.14 | kg |
| Water, GLO | river | 1.11E-04/13.56 | 1.25E-04/13.56 | 1.16E-04/13.56 | m3 |
| Zinc | river | 6.62E-09/6.40 | 7.45E-09/6.40 | 6.94E-09/6.40 | kg |
| Zinc | g.w., l.t. | 1.27E-04/35.17 | 1.44E-04/35.17 | 1.34E-04/35.17 | kg |
| Antimony | g.w., l.t. | 5.69E-06 | 6.41E-06 | 5.96E-06 | kg |
| Barium | g.w., l.t. | 3.18E-05 | 3.58E-05 | 3.33E-05 | kg |
| Barium | river | 4.93E-09 | 5.55E-09 | 5.17E-09 | kg |
| Beryllium | river | 6.31E-08 | 7.10E-08 | 6.61E-08 | kg |
| Beryllium | g.w., l.t. | 1.04E-04 | 1.17E-04 | 1.09E-04 | kg |
| Silver | river | 7.00E-12 | 7.89E-12 | 7.34E-12 | kg |
| Silver | g.w., l.t. | 1.40E-07 | 1.58E-07 | 1.47E-07 | kg |
| Titanium | g.w., l.t. | 1.85E-04 | 2.08E-0 | 1.94E-04 | kg |
| Titanium | river | 4.27E-08 | 4.81E-08 | 4.48E-08 | kg |
|  |  |  |  |  |  |
| *Waste to treatment* |  |  |  |  |  |
| Waste cement, hydrated \| | | 6.68E-02/1.48 | 4.51E-02/1.48 | 4.04E-02/1.48 | kg |

Legend: g.w.=ground water, l.t.=long term
